# Highly structured genetic diversity of *Bixa orellana* var. *urucurana*, the wild ancestor of annatto, in Brazilian Amazonia

**DOI:** 10.1371/journal.pone.0198593

**Published:** 2018-06-06

**Authors:** Gabriel Dequigiovanni, Santiago Linorio Ferreyra Ramos, Alessandro Alves-Pereira, Eliane Gomes Fabri, Doriane Picanço-Rodrigues, Charles Roland Clement, Paul Gepts, Elizabeth Ann Veasey

**Affiliations:** 1 Departamento de Genética, Escola Superior de Agricultura “Luiz de Queiroz”, Universidade de São Paulo (ESALQ-USP), Piracicaba, São Paulo, Brasil; 2 Instituto de Ciências Exatas e Tecnologia em Itacoatiara, Universidade Federal do Amazonas, Itacoatiara, Amazonas, Brasil; 3 Centro de Horticultura, Instituto Agronômico de Campinas, Campinas, São Paulo, Brasil; 4 Instituto de Ciências Biológicas, Universidade Federal do Amazonas (ICB-UFAM), Manaus, Amazonas, Brasil; 5 Instituto Nacional de Pesquisas da Amazônia (INPA), Manaus, Amazonas, Brasil; 6 Department of Plant Sciences, University of California, Davis, California, United States of America; National Cheng Kung University, TAIWAN

## Abstract

Annatto (*Bixa orellana* L.) is a tropical American crop, commercially valuable due to its application in the food and cosmetics industries as a natural dye. The wild ancestor of cultivated annatto is *B*. *orellana* var. *urucurana*. Although never cultivated, this variety occurs in open forests and anthropogenic landscapes, and is always associated with riparian environments. In this study, we evaluated the genetic diversity and structure of *B*. *orellana* var. *urucurana* populations in Brazilian Amazonia using 16 microsatellite loci. We used Ecological Niche Modeling (ENM) to characterize the potential geographical range of this variety in northern South America. We analyzed 170 samples from 10 municipalities in the states of Rondônia, Pará and Roraima. A total of 194 alleles was observed, with an average of 12.1 alleles per locus. Higher levels of expected (*H*_*E*_) than observed (*H*_*O*_) heterozygosities were found for all populations. Bayesian analysis, Neighbor-Joining dendrograms and PCAs suggest the existence of three strongly structured groups of populations. A strong and positive correlation between genetic and geographic distances was found, suggesting that genetic differentiation might be caused by geographic isolation. From species distribution modelling, we detected that South Rondônia, Madre di Dios River basin, Llanos de Mojos, Llanos de Orinoco and eastern Ecuador are highly suitable areas for wild annatto to occur, providing additional targets for future exploration and conservation. Climatic adaptation analyses revealed strong differentiation among populations, suggesting that precipitation plays a key role in wild annatto’s current and potential distribution patterns.

## Introduction

Annatto (*Bixa orellana* L.) is a tropical American crop [1], which probably originated in Amazonia [2–4]. Annatto is commercially valuable due to its application in the food and cosmetics industries, as a natural dye to be used instead of synthetic ones [5]. Five species are recognized in the genus *Bixa* (*Bixa orellana* L., *B*. *arborea* Huber, *B*. *excelsa* Gleason & Krukoff, *B*. *platycarpa* Ruiz & Pav. ex G. Don, and *B*. *urucurana* Willd.) [6,7], which belong to the Bixaceae family. The only cultivated species of the genus, *B*. *orellana*, is an evergreen shrub that is confined to the frost-free tropics [4,8]. An important distinction among the five species is growth habit, which can be either a tree or a shrub. *B*. *orellana* and *B*. *urucurana* are shrubs, while *B*. *arborea*, *B*. *excelsa* and *B*. *platycarpa* are trees [7,9]. Ducke [10] hypothesized that *B*. *excelsa* might have been the wild ancestor of *B*. *orellana*, which was accepted by Schultes [4] and Meyer et al. [11]. However, *B*. *excelsa* is a tree and it is unlikely that domestication during the Holocene would transform all known populations into a shrub [9]. Analysis of the domestication syndrome in the shrubby *Bixas* allowed Moreira et al. [9] to propose that *B*.*urucurana* is the wild ancestor of cultivated annatto, *B*. *orellana*. They also accepted Pilger’s proposal, published by Kuntz [12], that *urucurana* is a variety of *B*. *orellana* (*B*. *orellana* var. *urucurana* (Willd.) Kuntze ex Pilg.). The word ‘urucurana’ is derived from the Tupi language in which “rana” means false, and is often attributed to wild populations of a species with domesticated populations [9].

*B*.*orellana* var. *urucurana* occurs in open forests and anthropogenic landscapes, although never cultivated and is always associated with riparian environments [7,9]. While cultivated annatto always produces abundant pigment around its seeds, urucurana contains variable amounts. In areas where they co-exist, gene flow between them results in changes in pigment production, especially in the domesticated types [9]. The exact location where annatto was first domesticated is still unclear, mainly because of the wide distribution of variety *urucurana* in northern South America [9].

In order to make reasoned decisions about sampling procedures to preserve high levels of genetic diversity, researchers must know how genetic variation is organized and distributed throughout the geographic range of a species [13]. The assessment of genetic diversity and structure within and among populations of plants is generally performed using molecular markers. Microsatellites or SSRs (Simple Sequence Repeats) are among the most important molecular markers because they are abundant, co-dominant, with ample distribution in the genome, generally neutral and highly polymorphic [14]. Hence, SSR markers are important tools to assess genetic diversity and genetic structure of populations, especially for wild species [14]. There are very few genetic studies in annatto [15–17] and no studies have yet evaluated population structure and genetic diversity of wild populations of annatto (*B*. *orellana* var. *urucurana*).

In this study, 170 samples from 10 populations of wild annatto in Brazilian Amazonia were collected and analyzed using 16 SSR markers, in order to answer the following questions: a) what are the levels of genetic diversity in these populations?, b) what are the genetic relationships among these populations?, c) is genetic diversity geographically structured across these populations?, and d) what is the potential distribution of wild annatto in Amazonia? We used two approaches to answer these questions: neutral genetic variation (SSR markers) for questions ‘a’, ‘b’, ‘c’, and Ecological Niche Modeling (ENM) for question ‘d’ and how it may influence ‘a’, ‘b’ and ‘c’. ENM methods approximate a climatic envelope for the environmental requirements of a taxon from a set of its occurrence localities, summarizing environmental variation across those landscapes to develop a quantitative picture of the potential distribution of the species. They have provided a powerful tool for investigating the ecology and distribution of both plant and animal species [18,19], and their possible influences on patterns of genetic diversity of populations [20,21]. Therefore, ENM was utilized to characterize the potential geographical range of *B*. *orellana* var. *urucurana* in northern South America, based on these Brazilian Amazonian populations and also on online databases.

## Material and methods

### Plant material

**Fig 1:**
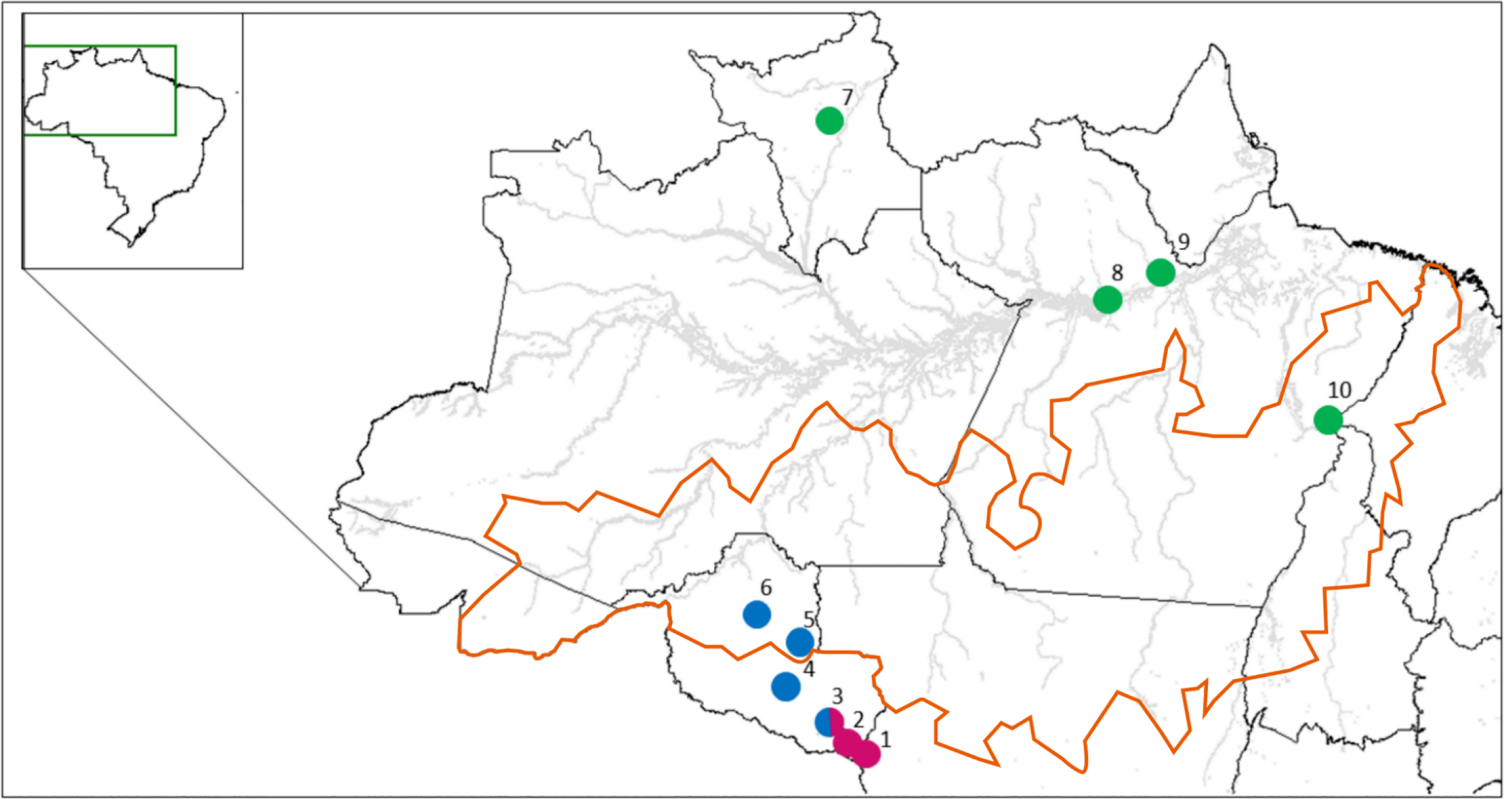
Geographic distribution of collection localities of 10 *Bixa orellana* var. *urucurana* populations and their assignments to the most likely number of clusters (*K* = 3) identified using Structure. Numbers represent collected populations: 1 –Cabixi, RO; 2 –Cerejeiras, RO; 3 –Corumbiara, RO; 4 –São Francisco do Guaporé, RO; 5 –Jí-Paraná, RO; 6 –Ariquemes, RO; 7 –Mucajaí, RR; 8 –Monte Alegre, PA; 9 –Almeirin, PA; 10 –Bom Jesus do Tocantins, PA. In orange, the delimitation of the arc of deforestation.

During our field work from 2009 to 2015, 170 plants of wild annatto (*Bixa orellana* var. *urucurana*) were located and collected in 10 municipalities in the states of Rondônia, Pará and Roraima, in Brazilian Amazonia (Table 1, Fig 1). A variety of seed bearing fruits with different shapes were observed during the field collections, and plants were usually associated with riparian environments (S1 Fig). From each plant, leaf samples were collected and stored in plastic bags containing silica gel. Some of the samples were stored in CTAB gels (3% (w/v) Cetyl Trimethyl Ammonium Bromide and 35% (w/v) NaCl). Each collection site was registered using Global Positioning System (GPS). No special permission was required for our sampling of annatto plants considering it was conducted according to the resolution 21, from CNPq (National Council for Scientific and Technological Development), in Brazil, which allows researchers to collect leaf samples for genetic analysis, as it is characterized as scientific research on phylogenetic relationships between geographic regions with annatto. Also, we have not accessed traditional knowledge related to wild annatto plants.

**Table 1:** Geographic location of the 10 populations of *Bixa orellana* var. *urucurana* collected in Brazilian Amazonia and used in this study, including sampling size (N), latitude and longitude (in decimal degrees).

Population ID / Municipality, State^a^	N	Latitude	Longitude
**1 –Cabixi, RO**	45	-13.48838	-60.60608
**2 –Cerejeiras, RO**	32	-13.17171	-60.80942
**3 –Corumbiara, RO**	26	-12.99158	-60.92277
**4 –São Francisco do Guaporé, RO**	9	-11.72616	-62.34804
**5 –Jí-Paraná, RO**	19	-11.49186	-62.41528
**6 –Ariquemes, RO**	18	-9.925150	-63.07129
**7 –Mucajaí, RR**	5	2.370000	-61.44000
**8 –Monte Alegre, PA**	5	-1.981198	-54.16811
**9 –Almeirin, PA**	4	-1.241724	-53.04789
**10 –Bom Jesus do Tocantins, PA**	7	-5.103889	-48.548889
**Total**	170	-	-

^a^ PA–Pará, RO–Rondônia, RR–Roraima

### DNA isolation, PCR amplification and genotyping of SSRs

Total genomic DNA was extracted from young leaves following Doyle and Doyle [22] with CTAB 3%. DNA concentration was determined by comparison with known concentrations of standard DNA (lambda DNA, Invitrogen) during electrophoresis in agarose gels (1% (w/v)) stained with GelRed (Biotium) under ultraviolet light.

Sixteen SSR markers developed for *B*. *orellana* [17,23] were used in this study (Table 2; S1 Table). Three fluorescent dyes (NED, FAM and HEX) were attached to the 5' end of the M13 universal primer sequence (5'- CACGACGTTGTAAAACGAC-3') following Schuelke [24]. Polymerase chain reaction (PCR) amplification of the DNA samples was done in a MyCycler Thermal Cycler (Bio-Rad), and performed in a final volume of 10 μL, consisting of 20 ng of DNA template, 1X PCR buffer (Fermentas, Vilnius, Lithuania), 0.25 mM of each dNTP, 1.5 mM of MgCl_2_, 2.5 pmol of forward and M13 labeled primers (FAM, HEX or NED dyes), 5 pmol of reverse primers and 1 U of *Taq* DNA polymerase (Fermentas).

**Table 2:** Genetic diversity estimates for 16 microsatellite (SSR) loci used to analyze wild annatto (*Bixa orellana* var. *urucurana*) collected in Brazilian Amazonia. Genetic diversity is described as number of alleles (*A*), observed (*H*_*O*_) and expected (*H*_*E*_) heterozygosities, and Shannon’s information index (*I*).

Loci	*A*	*H*_*O*_	*H*_*E*_	*I*
**BorA2**	5	0.354	0.438	0.712
**BorA3**	17	0.391	0.463	0.916
**BorA5**	12	0.401	0.525	0.968
**BorB1**	17	0.311	0.641	1.268
**BorB4**	17	0.352	0.609	1.111
**BorB5**	12	0.379	0.458	0.834
**BorB12**	14	0.421	0.508	1.015
**BorC5**	12	0.668	0.712	1.286
**BorD1**	8	0.357	0.423	0.752
**BorD2**	10	0.468	0.434	0.841
**BorF9**	12	0.231	0.629	1.220
**BorG4**	14	0.465	0.569	1.102
**BorG11**	19	0.426	0.679	1.354
**BorH3**	4	0.158	0.292	0.472
**BorH7**	5	0.494	0.443	0.692
**BorH10**	16	0.239	0.540	1.066
**Total**	194	-	-	-
**Mean**	12.125	0.382	0.522	0.975

PCR was carried out according to Schuelke [24] in a two-step process as follows: the first step consisted of an initial denaturing step of 94°C for 5 min, followed by 30 cycles of 94°C for 30 s, annealing temperature (Table 2) for 45 s, and 72°C for 45 s. The second step consisted of 8 cycles at 94°C for 30 s, 53°C for 45 s and 72°C for 45 s, and a final extension at 72°C for 10 min. Quality of PCR products was checked by electrophoresis in agarose gels (1.5% (w/v)) stained with GelRed (Biotium) under ultraviolet light. Capillary electrophoresis involved multiplexed marker panels, based on expected allele size, with two to three markers with at least 80 bp size differences. Fragment separation and detection were performed on an ABI Prism 3130xl capillary sequencer (Applied Biosystems) with the aid of GeneScan 500 ROX Size Standard (Applied Biosystems). DNA fragment sizes were determined using GeneMapper software (Applied Biosystems).

### SSR data analysis

Possible clusters of wild annatto were simulated using a Bayesian analysis with Structure software[25].The number of clusters (*K*) was estimated by performing ten independent runs for each *K* (from 1 to 10, the number of geographic locations, hereafter called populations for convenience), using 1,000,000 MCMC repetitions and a 200,000 burn-in period. Correlated allele frequencies and admixture were assumed. The most likely number of clusters was evaluated with the *adhoc* method of Evanno et al. [26].

Based on the original populations and the clusters identified by *Structure*, we estimated parameters for genetic diversity, including number of alleles per locus (*A*), effective allele number (Ne), allelic richness (*A*_*R*_) [27], observed (*H*_*O*_) and expected heterozygosity (*H*_*E*_), in addition to Wright’s [28] inbreeding coefficient (*f*). The apparent outcrossing rate (${\hat{t}}_{a}$) was estimated considering the inbreeding coefficient (*f*) for each population [29], so that ${\hat{t}}_{a}$ = (1-*f*)/(1+*f*). The genetic diversity indices *A*, Ne, *H*_*O*_ and *H*_*E*_ were estimated with GenAlEx 6.5 [30], and estimations of *A*_*R*_ and *f*, with confidence intervals based upon 1,000 bootstrap replicates, were obtained with diversity [31] and poppr [32] for R [33].

In order to represent the relationships between individuals and populations, neighbor-joining [34] dendrograms were constructed with Phylip 3.5 [35], based on Cavalli-Sforza and Edwards’chord distance (*D*_*CE*_) [36] obtained with MSA 4.05 [37]. The chord distance is a geometric distance and performs well for the reconstruction of relationships among populations [38]. Confidence of relationships was assessed with 1,000 bootstrap replicates. Final trees were formatted in FigTree 1.4.1 (http://tree.bio.ed.ac.uk/software/figtree/). A principal coordinate analysis was used to visualize the dispersion of samples as a function of genetic variation using GenAlEx 6.5 [30].

Hierarchical distribution of genetic variation within and among populations of wild annatto, and within and among groups according to the Structure analysis was evaluated using “locus-by-locus” AMOVA with GenAlEx 6.5 [30]. Gene flow (*N*_*m*_) among populations was estimated by calculating *N*_m_ = (1—*F*_*ST*_)/4*F*_*ST*_ [39]. In addition, the Mantel test was used to evaluate the correlation between Nei’s genetic distance and geographic distance (km) among populations using Adegenet [40] for R [33]. Significance was assessed by conducting 9,999 permutations.

### Potential distribution of *B*. *orellana* var. *urucurana*

The potential distribution of *B*. *orellana* var. *urucurana* was estimated using the maximum entropy algorithm of Maxent v. 3.3.3e [41]. Maxent estimates the potential distribution of a taxa from a maximum entropy probability distribution using presence-only data [42]. The resulting model is a geographical projection of habitat suitability for the target species where values close to 0 indicate sites that do not match with the niche requirements of the species, and values close to 1 indicate sites that fully match the niche requirements. A total of 184 presence-only records were compiled from field work and from georeferenced herbarium data extracted from the speciesLink project (http://splink.cria.org.br) and Global Biodiversity Information Facility (GBIF) portal. All geographic coordinates were manually verified and incomplete or imprecise records were discarded. For each occurrence record, we obtained 19 bioclimatic variables derived from monthly temperature and rainfall from the WORLDCLIM database with resolutions of 2.5’ [43]. The 19 bioclimatic variables are: BIO1 = Annual Mean Temperature; BIO2 = Mean Diurnal Range (Mean of monthly (max temp—min temp)); BIO3 = Isothermality (BIO2/BIO7) (* 100); BIO4 = Temperature Seasonality (standard deviation *100); BIO5 = Max Temperature of Warmest Month; BIO6 = Min Temperature of Coldest Month; BIO7 = Temperature Annual Range (BIO5-BIO6); BIO8 = Mean Temperature of Wettest Quarter; BIO9 = Mean Temperature of Driest Quarter; BIO10 = Mean Temperature of Warmest Quarter; BIO11 = Mean Temperature of Coldest Quarter; BIO12 = Annual Precipitation; BIO13 = Precipitation of Wettest Month; BIO14 = Precipitation of Driest Month; BIO15 = Precipitation Seasonality (Coefficient of Variation); BIO16 = Precipitation of Wettest Quarter; BIO17 = Precipitation of Driest Quarter; BIO18 = Precipitation of Warmest Quarter; BIO19 = Precipitation of Coldest Quarter.

Fifteen model replicates were run with 75% of occurrences used for calibration and different subsets (25%) used for validation. A logistic threshold value of 10 percentile training presence was retained to separate climatically favorable areas from marginally fit areas. The accuracy of model prediction was evaluated using the area under the curve (AUC), where 1 was the maximum prediction and 0.5 suggested a random prediction [44]. Permutation procedure was used to define contributions of the variables to the models.

Because we observed a high correlation between genetic and geographic distances, we tested the assumption that most of the variability is due to environmental factors.To compare the environmental characteristics of the different areas, we performed principal components analysis (PCA) with ade4 [45] for R [33].

## Results

### Genetic diversity

All the 16 SSR markers were polymorphic, with a total of 194 alleles. The number of alleles per locus ranged from four (BorH3) to 19 (BorG11) with an average of 12 alleles per locus (Table 2). The observed heterozygosity (*H*_*O*_) ranged from 0.158 to 0.712 across loci, with a mean of 0.385, while the expected heterozygosity (*H*_*E*_) ranged from 0.292 to 0.679, with a mean of 0.520. All loci had heterozygote deficits greater than 10%. The mean Shannon diversity index (*I*) was 0.975, ranging from 0.472 to 1.354 (Table 2).

Genetic diversity estimates of the 10 populations showed a mean number of alleles per locus (*Ā*) of 3.86 (Table 3). Forty-one private alleles were observed, representing 21% of all alleles. The population from Bom Jesus do Tocantins (Population 10 in Table 1 and Fig 1) showed the highest number of private alleles (15) (Table 3). The mean values of observed (*H*_*O*_) and expected (*H*_*E*_) heterozygosities for all populations were 0.382 and 0.522, respectively. Significant inbreeding coefficients (*f*) were detected in most populations, ranging from 0.047 to 0.565. The mean apparent outcrossing rate (${\hat{t}}_{a}$) was 0.609. When disregarding the populations with small sampling sizes, such as Monte Alegre (N = 5), Mucajaí (N = 5) and Almeirim (N = 4), the mean value of this parameter increased to 0.690.

**Table 3:** Genetic diversity estimated for 10 populations of *Bixa orellana* var. *urucurana*, including mean number of alleles per locus (*Ā*), alellic richness (*A*_*R*_), mean number of effective alleles per locus (*N*_*E*_), observed (*H*_*O*_) and expected (*H*_*E*_) heterozygosities, inbreeding coefficient (*f* = 1—*H*_*O*_*/H*_*E*_), and apparent outcrossing rate (${\hat{t}}_{a}$).

Population, State^a^	*Ā*_(private aleles)_	*A*_*R*_	*N*_*E*_	*H*_*O*_	*H*_*E*_	*f*	${\hat{t}}_{a}$
**1.Cabixi, RO**	5.813 (1)	1.582	3.000	0.449	0.577	0.217*	0.643
**2.Cerejeiras, RO**	4.875 (1)	1.558	2.845	0.442	0.554	0.181*	0.693
**3.Corumbiara, RO**	4.750 (1)	1.577	3.127	0.445	0.571	0.196*	0.672
**4.S. F. do Guaporé, RO**	3.625 (3)	1.564	2.512	0.437	0.548	0.168*	0.712
**5. Jí-Paraná, RO**	5.000 (3)	1.645	3.379	0.426	0.636	0.309*	0.528
**6. Ariquemes, RO**	3.875 (3)	1.565	2.566	0.453	0.553	0.166*	0.715
**7. Mucajaí, RR**	3.438 (9)	1.631	2.816	0.268	0.596	0.565*	0.278
**8. Monte Alegre, PA**	1.813 (2)	1.338	1.653	0.313	0.321	0.047	0.910
**9. Almeirin, PA**	2.063 (3)	1.353	1.865	0.146	0.327	0.508*	0.326
**10. B.J.Tocantins, PA**	3.313 (15)	1.535	2.596	0.471	0.516	0.081*	0.850
**Mean**	3.856	1.535	2.636	0.385	0.519	0.243	0.609

^a^ PA–Pará, RO–Rondônia, RR—Roraima

* Significant based upon 1,000 bootstrap replicates

### Genetic structure

**Fig 2:**
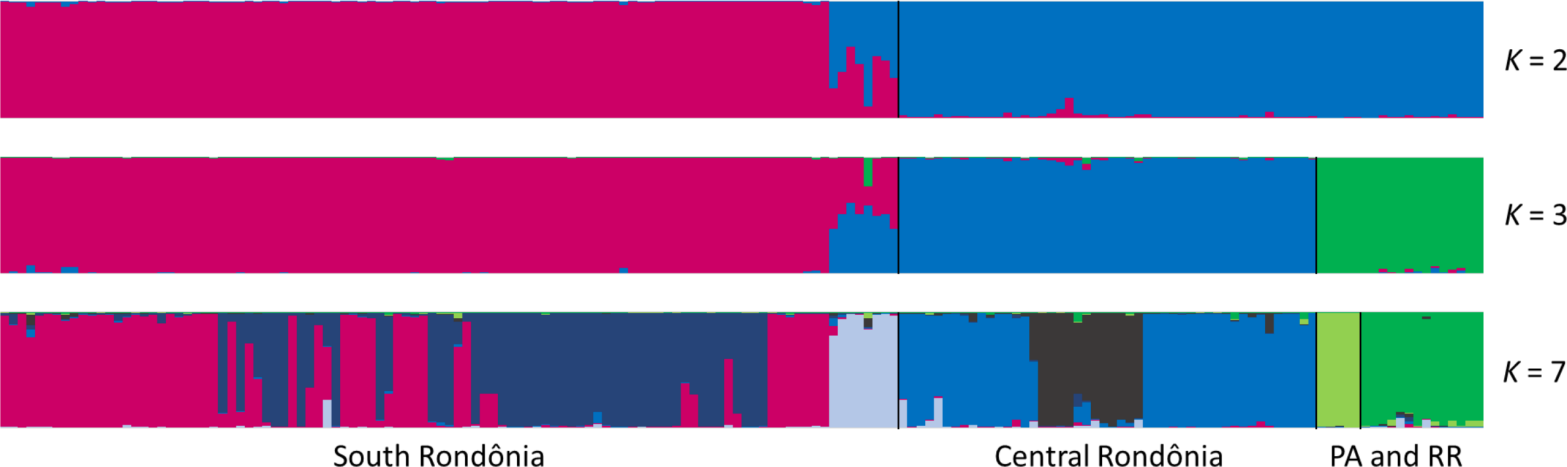
Assignment of each of 170 *Bixa orellana* var. *urucurana* plants collected in Brazilian Amazonia to groups simulated by *Structure* at *K* = 2, *K* = 3 and *K* = 7 based on 16 SSR loci.

The 170 wild annatto plants of 10 populations were grouped into genetic clusters by the Structure simulations, with a clear Δ*K* maximum at *K* = 3, and possible subtructure at *K* = 2 and *K* = 7 (Fig 2, S2 Fig). According to *K* = 3, group I (hereafter South RO) included the populations from Cabixi, Cerejeiras and Corumbiara in the Guaporé River basin in southern Rondônia State. Group II (hereafter Central RO) included the populations from Ariquemes and Jí-Paranain the Jí-Parana River basin, and São Francisco do Guaporé, from the Guaporé River basin, located in central Rondônia State. The groups of South RO and Central RO meet and mix at Corumbiara, in southern Rondônia. Group III (hereafter PA and RR) included all the other populations, both north of the Amazon River in Roraima and Pará, and south of the Amazon River in eastern Pará (Fig 1). At *K* = 2, South RO was allocated in one group and Central RO was clustered together with populations from PA and RR. At *K* = 7, the PA and RR group was subdivided, with the north of the Amazon River in one group, and eastern Pará in another group; groups South RO and Central RO were also subdivided, confirming the high diversity observed within bothgroups (Table 3). Also, a separate group was formed at Corumbiara, in southern Rondônia, where we found mixed populations from South and Central Rondônia at both *K* = 2 and *K* = 3 (Fig 2).

**Fig 3:**
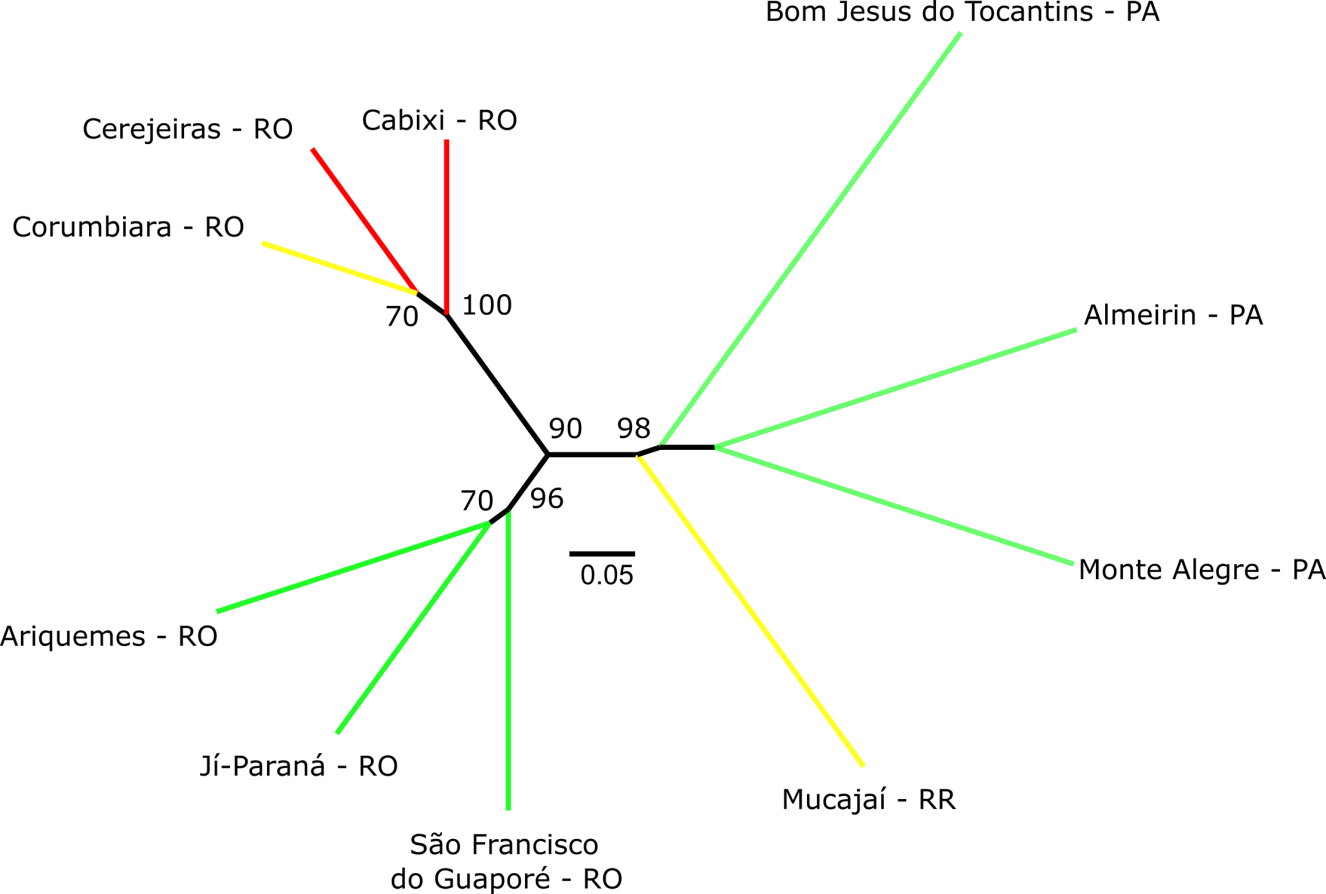
Unrooted neighbor-joining dendrogram of 10 *Bixa orellana* var. *urucurana* populations collected in Brazilian Amazonia based on Cavalli-Sforza & Edwards chord distance [36] estimated from 16 SSR. Colors are according to the probability of occurrence of *Bixa orellana* var. *urucurana* simulated by Ecological Niche Modeling (Fig 5). Red = very high probability, yellow = high probability and green = moderate probability.

Relationships among populations in the dendrogram (Fig 3) generally agreed with *Structure* and PCoA (not shown data). The relationship among individuals in the dendrogram (S3 Fig) also agreed with *Structure* and PCoA results. In the dendrogram of individual plants (S3 Fig), the Central Rondônia populations have a slightly greater relationship with the non-Rondônia populations, rather than with the South Rondônia populations, suggesting a difference that may be due to adaptation to the more savanna-like climate of South Rondônia.

According to Mantel’s test, 74% of the genetic divergence among populations was due to the geographic distances among them. A strong and positive correlation between genetic and geographic distances (r = 0.860, p = 0.003) suggests that genetic differentiation among the 10 populations is due to isolation by distance, not surprising given the long distances between Rondônia and Roraima, especially.

AMOVA revealed that 21% of the genetic variation was among populations, and the remaining 79% was within populations (p = 0.000), showing that, although most of the diversity is within populations, the variation due to sub-division of the populations is significant (Table 4). The microsatellite data also showed low levels of gene flow among populations (*N*_*m*_ = 0.545). However, when analyzed by the *a priori* populations, we observed an average gene flow of 1.878 among populations from Rondônia, while the other regions presented lower values (*N*_*m*_ = 0.65). The populations from south Rondônia separately showed a high gene flow amongthem (*N*_*m*_ = 4.843).

**Table 4:** Analysis of molecular variance (AMOVA) performed for 16 SSR and 170 *Bixa orellana* var. *urucurana* plants collected in Brazilian Amazonia.

Source of variation	Degrees of freedom	Sum of squares	Mean square	Variance	%
**Among populations**	9	415.758	46.195	1.301	21
**Within populations**	330	1611.165	4.882	4.882	79
**Total**	339	2026.924		6.183	100

### Species distribution modelling and climatic adaptation

**Fig 4:**
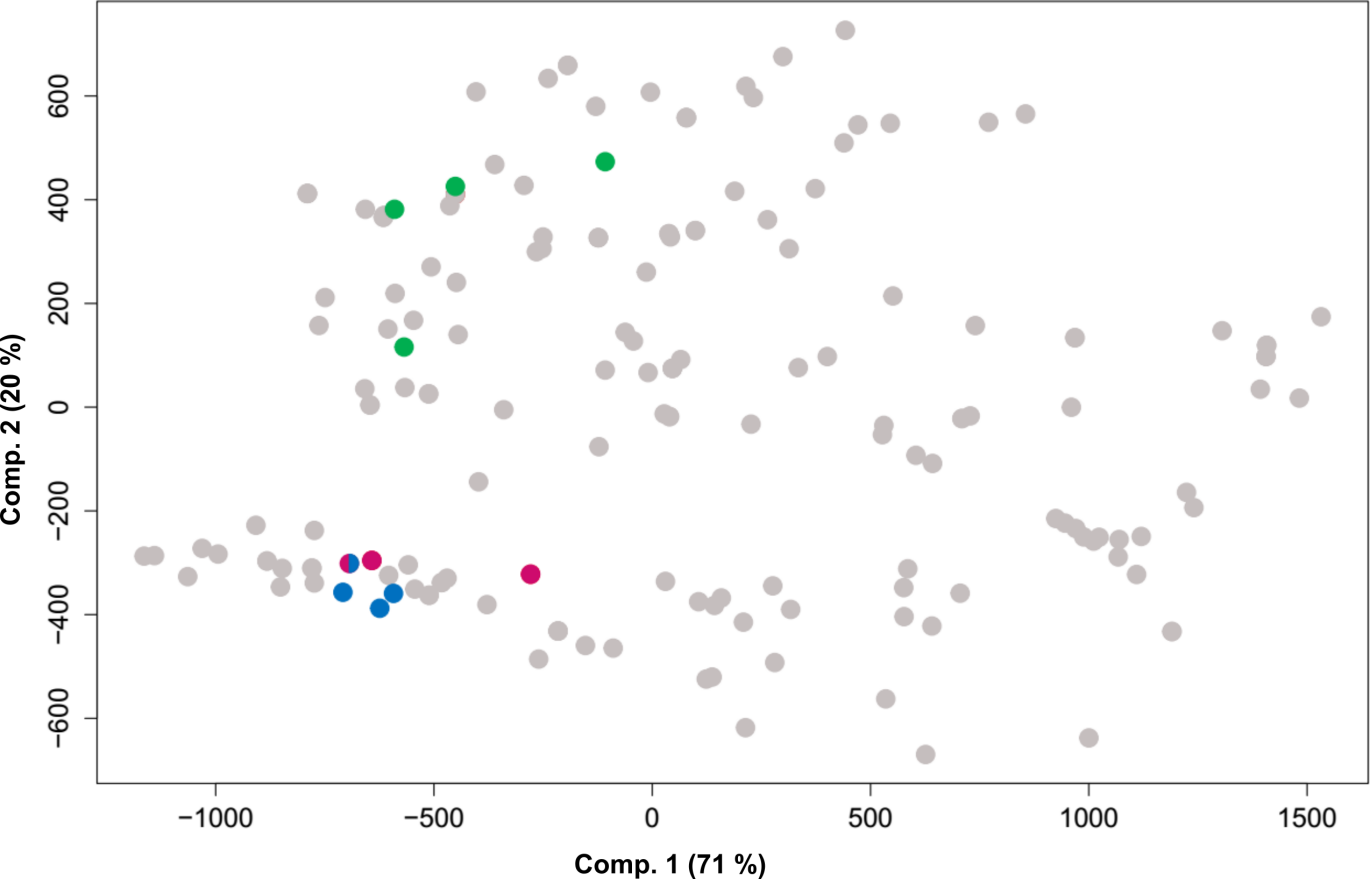
Principal component analysis (PCA) performed on 19 bioclimatic variables extracted from the Worldclim database for 10 wild annatto (*Bixa orellana* var. *urucurana*) populations sampled and 174 presence-only records from online databases. Colors are according to the Structure analysis at *K* = 3 and online databases: Pink = South Rondônia accessions; Blue = Central Rondônia accessions; Green = accessions from the states of Pará and Roraima; and Gray = online databases occurrences.

The result of the PCA using the 10 populations sampled in this study and 174 presence-only records with the 19 bioclimatic variables generated three main components that explained more than 91% of the variation. Graphical representation of climate space associated with the first two PCA axes revealed high climatic differentiation between populations from Rondônia, and Pará and Roraima along the second PCA axis (Fig 4). There was also a moderate degree of climatic overlap between Central Rondônia and South Rondônia groups, indicating evidence of incomplete separation between the Rondônia groups according to the bioclimatic variables.

The relative contributions of climatic variables to the PCA axes show that niche differentiation along Components 1 and 2 was driven primarily by precipitation requirements (S2 Table). Principal component 1 (PC1) represented 71% of the variation and was mostly explained by variable Bio12 (Annual Precipitation). The annual precipitation varied from 1,309 mm (on the left) to 3,644 mm (on the right). Our sampled populations presented annual precipitations ranging from 1,669 mm to 2,192 mm. While variable Bio12 contributed positively, variable Bio15 (Precipitation Seasonality) contributed negatively in the first axis. On the other hand, the second axis explained 20% of the variation and variable Bio19 (Precipitation of Coldest Quarter) was the most informative variable in this axis (S2 Table), ranging from 87 mm (on the top) to 1,388 mm (on the bottom). Our sampled populations ranged from 94 to 917 mm of precipitation in the coldest quarter. Variables Bio16 (Precipitation of Wettest Quarter) and Bio17 (Precipitation of Driest Quarter) also played important roles in the analysis.

**Fig 5:**
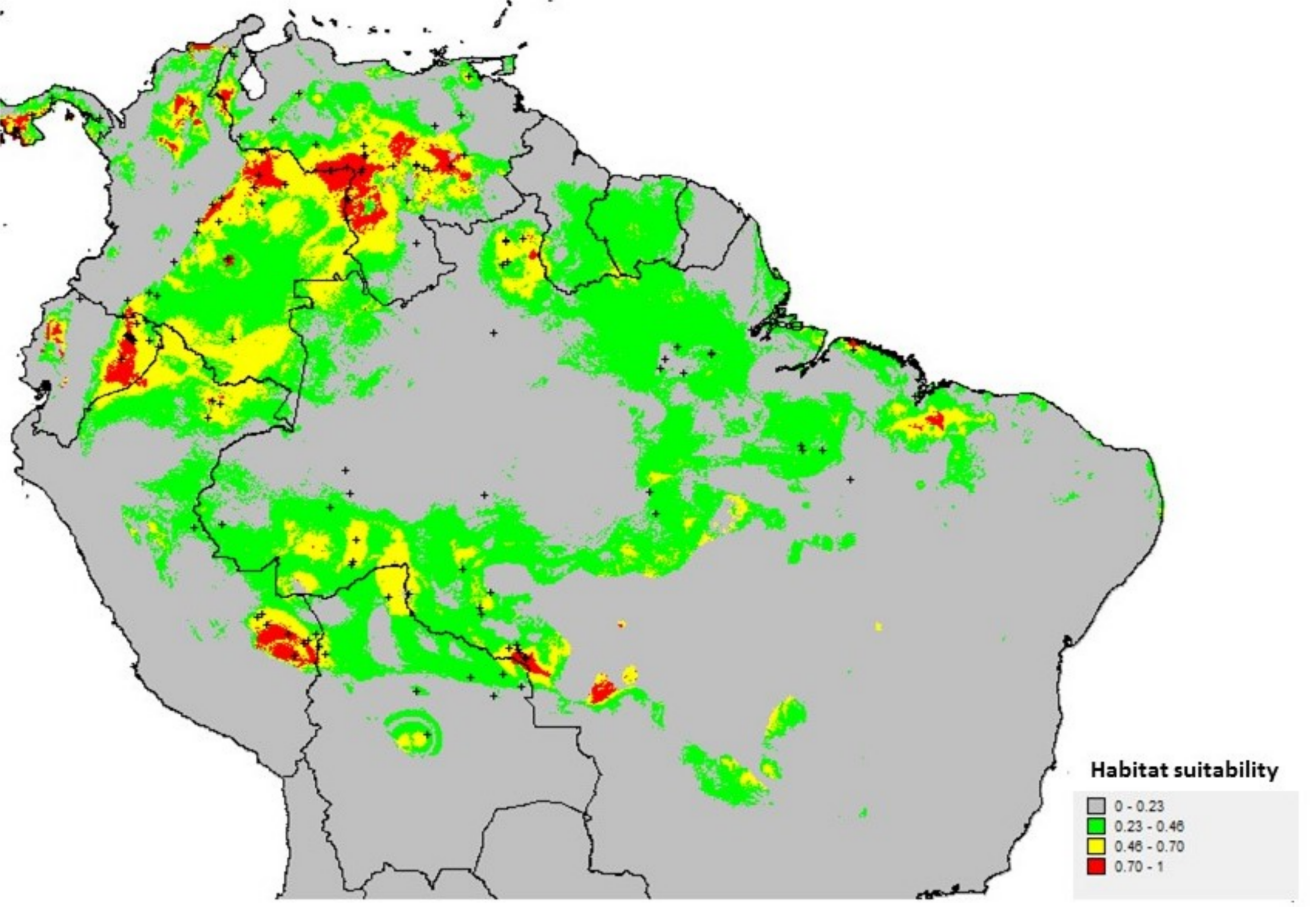
Potential distribution as probability of occurrence of *Bixa orellana* var. *urucurana* simulated by Ecological Niche Modeling. Black crosses are the presence records used for the simulation.

Over 15 replicate runs, the potential distribution of *B*. *orellana* var. *urucurana* was estimated with a high area-under-the-curve (AUC) value (0.941) implying very low rates of false negative and positive suitability predictions (Fig 5). The climate envelope of wild annatto is largely determined by precipitation, and the most important variables for the model were Bio19 (Precipitation of coldest quarter, 23.5%), Bio13 (Precipitation of wettest month, 12.4%) and Bio12 (Annual precipitation, 11.5%). Temperature seasonality (Bio4, 17.5%) also plays a substantial role in the niche.

## Discussion

### Genetic diversity

This is the first genetic study with wild populations of annatto. The levels of heterozygosity averaged over all loci (*H*_*E*_ = 0.520; *H*_*O*_ = 0.385) among the 10 populations were considerably higher than those in a germplasm bank (*H*_*E*_ = 0.470; *H*_*O*_ = 0.170) with 63 cultivated varieties of annatto using the same 16 SSR loci [46]. It is expected that wild populations exhibit higher levels of diversity in relation to cultivated populations, as the latter have passed through a domestication bottleneck [47] and generally further bottlenecks due to distribution and diversification [48], including different selection pressures for yield [49].

The high levels of inbreeding coeficient (*f*) observed in our study, the estimated apparent outcrossing rate (${\hat{t}}_{a}$ = 0.609) and the apparent selfing rate (0.391) indicate a mixed mating system for wild annatto. The existence of crosses between related individuals increases homozygosity [50]. These results are in agreement with other studies on cultivated annatto, such as those obtained by Rivera-Madrid et al. [51], who conducted controlled pollinations in annatto accessions in an experimental field in Mexico, suggesting that annatto can tolerate both types of pollination, showing cross-pollination values of 57% and self-fertilization of 31%. Vilares et al. [52] also concluded that there is natural selfing in annatto. Valdez-Ojeda et al. [16] found high multilocus outcrossing rates (0.75) based on 50 SRAP markers and concluded that *B*. *orellana* has a mixed mating system. A recent study compared the mating system of annatto under different agronomic systems in Mexico using SRAP markers [51], showing a mixed mating system for annatto cultivated in backyards, while those cultivated under polyculture (milpa system) and monoculture systems showed predominantly outcrossing mating systems. Considering that most of the above results have shown mixed mating system for cultivated annatto, we may conclude that domestication of annatto did not include modifications in the mating system.

### Genetic structure

Plant populations are not randomly arranged assemblages of genotypes but are structured in space and time. Genetic structure results from the action of migration, mutation, selection, and drift, that operates within the historical and biological context of each plant species [13]. In this study, most of the genetic variability was observed within populations (79%), as shown in the AMOVA results. However, the high value of *F*_*ST*_ (0.201) indicates the existence of strong structure among populations. Dick et al. [53] reviewed the genetic structure among populations of 42 different tropical species separated by more than 50 km and found high levels of population differentiation (*F*_*ST*_ = 0.177). This may be due to the fact that tropical species are experiencing moderate to high levels of inbreeding, as a result of the association between low population density, density-dependent animal pollination, and mixed mating systems, factors that may be restricting gene flow [53].

The mean level of gene flow among *B*. *orellana* var. *urucurana* populations was low in this study (*N*_*m*_ = 0.545), since many of the populations were very distant apart, especially those from Rondônia and the ones from Roraima and Pará, but also between those from Roraima and Pará. According to Wright [54], a migration rate of *N*_*m*_ = 1.0 is theoretically necessary to counteract the effect of genetic drift. In this sense, our results suggest that genetic drift may have been a dominant factor determining the genetic structure of *B*. *orellana* var. *urucurana* populations. Gene flow among wild annatto populations may occur by seed dispersal along riversand streams [9], but also through cross-pollination by bees [55]. All wild annatto populations we found in Amazonia occurred in open forests and anthropogenic landscapes, although never cultivated, and always associated with riparian environments, suggesting that gene flow among distant populations may have occurred by the transport of fruits, and consequently seeds, along rivers. The differentiation between the Rondônia and the Pará/Roraima populations is also due to the fact that the distances among these populations are very large, in addition to the existence of many barriers that can prevent or slow gene flow (see discussion below). In fact, what is surprising is that the Amazon River did not prove to be a major barrier between the northern populations (Roraima and northern Pará) and the southeastern Pará population until *K* = 7.

The structure of the Rondônia populations in two groups may occur because the southern populations of this state (Cabixi, Corumbiara and Cerejeiras) are in the Guaporé River basin, while the populations from the center of Rondônia state (Ariquemes and Jí Paraná) are in the Jí-Paraná River basin, so that gene flow by the transport of seeds along the rivers between these populations is infrequent. However, mixed populations between the two groups were found at Corumbiara, in southern Rondônia, classified as a separate group at *K* = 7, indicating an intermediate area with a higher gene flow between the two groups.

The significant correlation values obtained between genetic and geographic distances indicate isolation by distance for the wild annatto populations. In the cluster analysis, based on Cavalli-Sforza and Edwards [36] chord distance and the neighbor-joining method, as well as in the PCoA and the Bayesian analysis, three genetically distinct and consistent groups were identified. The groups were formed based on the geographical location of the collected populations. Carvalho et al. [56], using isozymes, also found that genetic differences in cultivated annatto accessions correspond to distinct geographical locations. However, Medina et al. [57] evaluated 36 genotypes of cultivated annatto collected in Venezuela and Brazil, also using isozymes, and found no correlation between geographic and genetic distances. The explanation given by the authors was the anthropogenic influence in this crop´s cultivation. Menezes et al. [58] found similar patterns to those obtained in this study when assessing wild cotton (*Gossypium mustelinum* Miers) in the state of Bahia, the only cotton species native to Brazil. These authors found high correlation between the genetic and geographic distances using Mantel’s test (r = 0.87, p = 0.05).

Our results provide relevant information for conservation of annatto germplasm. Wild annatto populations may be a source of new alleles, which might be useful to increase the genetic basis of annatto in breeding programs and for conservation strategies. According to Moreira et al. [9] and also our field observations, when wild annatto grows near commercial annatto fields, farmers tend to remove the existing wild types, because they naturally cross, generating production losses in the progenies. As occurrences of wild annatto are mainly in the peripheries of Amazonia, and in most cases, in anthropogenic areas [9], these practices may lead to a decrease of wild annatto populations, fragmentation of the native habitat of the species, and overall genetic diversity of the species.

### Distribution and climatic adaptation of wild annatto populations

The high global *F*_*ST*_ value (0.201), identifying strong structure among populations, is partially due to our sampling effort, but may also reflect the real and modeled distribution of wild annatto. According to the ENM, wild annatto is not uniformly distributed throughout Amazonia, with a large area in Central Amazonia (Fig 5) unsuitable for the species. If this ENM is accurate, it may be very difficult to find large natural populations of wild annatto in this wide area. This fragmented distribution may be a major barrier for gene flow among populations, which may explain the high genetic differentiation and also the pattern of isolation by distance suggested by the Mantel test. This model also helps explain the lack of observation of wild annatto in these areas during our field collections, or the observation of smaller populations, and the low number of herbaria registers. Comparing the Southern Rondonia populations with the Central Rondonia populations, there were an abundance of plants to be sampled in Southern Rondônia, while the wild annatto populations found in Central Rondônia had fewer plants. As a matter of fact, we collected all of the plants from the center of Rondônia populations that we came across with. South Rondônia is an area of high suitability for wild annatto (Fig 5), according to the ENM, which may explain the higher sampling obtained in this area.

In addition, we may consider that the high divergence among populations may be due to what is known as the “arc of deforestation”, a region where the agricultural frontier advances towards the forest [59]. There are 500 thousand km^2^ of land that goes from the east and south of Pará towards the west, passing through the States of Mato Grosso, Rondônia and Acre (Fig 1). The arc of deforestation has the highest rates of deforestation of the Amazon forest, which is also probably causing the fragmentation of wild annatto populations. The possible occurrence of small isolated populations of wild annatto across Amazonia also agrees with the higher levels of intrapopulation inbreeding coefficients, suggesting the action of genetic drift coupled with inbreeding within the populations included in this study.

Temperature and precipitation are considered as major factors in determining species distributions [60]. Our ENM model suggests that precipitation plays a key role in wild annatto’s current and potential distribution pattern. In general, favourable habitats are drier or seasonally drier areas, which suggest the species tolerance to drought conditions, even though we observe a wide variation in levels of precipitation in the regions with occurrence of the species. Although temperature variables did not contribute much to the distribution of the species, we observed a wide variation in temperature averages. The large intervals of temperature and precipitation suggest that the species has ample adaptation. Temperature and precipitation have been identifed as major selective pressures driving plant adaptation because they are very important for plant growth, development, and reproduction [61,62]. Adaptation to new habitats is also a potential plant response to shifts in environmental conditions, which is also crucial in the context of climate change [63].

Not surprisingly, the potential distribution coincided approximately with the current occurrence reported in online distribution databases, but also suitable localities were predicted outside the presently known range of the species. These localities could be targeted with field surveys that might identify unknown populations. However, a considerable number of occurrences are in very low probability areas, suggesting that this large-scale analysis did a poor job of capturing urucurana’s adaptation to riparian conditions in drier climates. The species is mostly confined to the periphery of Amazonia, but also to areas in the drier parts of western Central America. According to Clement et al. [3], the periphery of Amazonia appears to be the area where the majority of Amazonian crops were domesticated. The upper Madeira River basin, in southwestern Amazonia, is an important part of the periphery and has been recognized as a probable region of crop origins for some time [64]. Levis et al. [65] also found higher abundance and richness of domesticated species in southwestern Amazonia.

Piperno and Pearsall [66] also highlighted the importance of the periphery, mainly in extreme northwestern Amazonia and the adjacent Llanos of the Orinoco River basin, the Guiana shield, as well as in southwestern Amazonia, especially the Llanos de Mojos, in Bolivia. The potential distribution map predicted moderately suitable habitat in the Llanos de Mojos. The Llanos de Mojos is a tropical savanna in Bolivian Amazonia, shaped by cycles of drought and flood [67]. This grassland environment presents a 2- to 7-month dry season and a total annual rainfall varying between 1,500 and 1,800 mm. The dry season lasts from May through September, when weeks pass without precipitation [67]. Complex societies inhabitated this region at the time of the European conquest, and managed dozens of species, leading Clement [68] to propose a micro-center of diversity of crop genetics resources in Llanos de Mojos. Also, the only archaeological record of annatto in Amazonia comes from this area, and is dated to 2,400 years before present [69].

The Madre de Dios Basin is also a highly suitable area for *B*. *orellana* var. *urucurana*. According to Leal and Clavijo [70], the genus *Bixa* probably originated between the Huallaga-Ucayali River, and the Madre de Dios-Madeira River, along the slopes of the eastern Andes. The Madre de Dios River joins with the Mamore River to become the Madeira River, also an important area for crop domestication [64]. The Madre de Dios Basin drains an area of approximately 90,000 km along the eastern flank of the Andes in southeastern Peru, ranging in elevation from 200 m to over 4,000 m [71]. The vegetation is predominantly evergreen or semi-evergreen forest [72]. It presents a humid tropical climate with annual rainfall varying from 1,200 mm to 3,300 mm, generally increasing from east to west, and the rainy season occurring from October to April [72].

We also found highly suitable habitat for wild annatto in the Llanos del Orinoco, in western Venezuela and northeastern Colombia. This is an area of extensive plains, covered mainly by savanna vegetation. This ecoregion has a strongly seasonal climate, with a single dry season extending between November and May, and a single rainy season between May and October. The temperature prevailing in these tropical American lowlands is macrothermic, with mean anual temperatures ranging from 26°C to 28°C and monthly average maximum temperatures between 34°C and 37°C. The rainfall of the Llanos region shows a regime characterized by very pronounced differences during the months of the year, with annual rainfall ranging from 850 mm to 1,800 mm [73].

The areas of the Llanos de Orinoco, Llanos de Mojos, Madre de Dios and also South of Rondônia have very similar climatic characteristics, which make all of them areas suitable for the occurrence of the species. These are drier or seasonally drier areas, and are located in the peripheries of the Amazon, consistent with the favorable areas identified by Moreira el al. [9]. In Rondônia, our sampled populations in the savannas of South Rondônia are in an area of very high probability in the potential distribution map, while Central Rondônia populations are in an area with much less probability, and this may suggest differential adaptation.

On the other hand, an interesting result of the ENM model was the high probability area in eastern Ecuador.The eastern lowlands in Equador experience abundant rainfall, sometimes exceeding 5,000 mm per year and mean temperatures ranging from 25°C to 28°C. These findings also suggest adaptation of *B*. *orellana* var. *urucurana* to different niches.

According to our ENM model, we noticed that the distribution of our wild annatto sampling was made in quite marginal populations (suitability for Central Rondônia, Pará and Roraima is less than 0.7) and therefore, the picture of the genetic diversity is quite partial. We also observed that in the area between the two regions, there are no predicted populations (low suitability). Therefore, the gene flow among these two different regions could not be recent, but historical, and also probably due to some other historical factors of the populations. We do not know if there are populations in Ecuador that could clarify the relationship among the two main regions studied. The ENM model results in this study implies the recommendation that further collection expeditions should be made sampling *B*. *orellana* var. *urucurana* populations from eastern Ecuador, western Venezuela, northeastern Colombia and the Llanos de Mojos, Bolivia, as well as the State of Mato Grosso and Northeast Brazil.

## Conclusions

The microsatellite loci used in this study revealed high levels of genetic diversity in populations of wild annatto and this diversity is highly structured according to the geographic origin of populations. Wild annatto appears to have a mixed mating system, which may contribute to the patterns of genetic structure observed. Our map of the potential distribution of the species allowed the identification of other potential areas of occurrence in Amazonia and in northern South America. Interestingly, our ENM predicted a wide area of low suitability for wild annatto across Central Amazonia. This predicted occurrence plus increasing population fragmentation resulting from Amazonia deforestation contribute to the low genetic connectivity among disjunct populations of wild annatto. Therefore, our study demonstrates how ecological and anthropic factors may have an impact on the genetics of a native Amazonian species. New plant collections will add to a better understanding of the genetic diversity and structure of wild annatto, as well as the understanding of the crop’s domestication from these wild populations.

## Supporting information

S1 Table SSR data for each *Bixa orellana* var. *urucurana* individual and populations. (TXT)

S2 Table Factor loadings of principal component analysis (rotation) on a set of 19 bioclimatic variables retained for their contribution to the model of distribution (higher values in bold characters). (DOCX)

S1 Fig Morphological variation on fruits and plant architecture of wild annatto (*Bixa orellana* var. *urucurana*). (TIFF)

S2 Fig Evanno et al. (2005) plot detecting the number of *K* groups that best fit the data for *Bixa orellana* var. *urucurana* individuals assessed with 16 SSR loci. (TIFF)

S3 Fig Neighbor-joining dendrogram for individuals based on Cavalli-Sforza and Edwards (1967) chord distance estimated from 16 nuclear microsatellites (SSR) and 170 wild annatto (*Bixa orellana* var. *urucurana*) accessions. Branches are colored according to the Structure simulation for *K* = 3.
(TIFF)

## References

[1] Arce J. El achiote *Bixa orellana* L. cultivo promisorio para el trópico. Earth. first ed. 1999 p. 149.

[2] Sandy-Cuen PM, Becerra R. Manejo campesino de recursos naturales. El achiote. BioDiversitas. 2003;7: 7–11.

[3] Clement CR, de Cristo-Araújo M, D’Eeckenbrugge GC, Alves Pereira A, Picanço-Rodrigues D. Origin and Domestication of Native Amazonian Crops. Diversity. Molecular Diversity Preservation International; 2010;2: 72–106. doi: 10.3390/d2010072

[4] Schultes R. Amazonian cultigens and their northward and westward migrations in pre-Columbian times In: Stone D, editor. Pre-Columbian Plant Migration Papers of the Peabody Museum of Archaeology and Ethnology. 1st ed. Cambridge: Harvard University; 1984 pp. 19–38.

[5] Nisar N, Li L, Lu S, Khin NC, Pogson BJ. Carotenoid metabolism in plants. Molecular Plant. 2015 pp. 68–82. doi: 10.1016/j.molp.2014.12.007 25578273 10.1016/j.molp.2014.12.007

[6] The Plant List Version 1.1. In: Published on the internet [Internet]. 2013. Available: http://www.theplantlist.org/

[7] Baer D. Systematics of the genus Bixa and geography of the cultivated annatto. Dissertation. University of California—Los Angeles. 1976.

[8] Akshatha V, Giridhar P, Ravishankar GA. Morphological diversity in *Bixa orellana* L. and variations in annatto pigment yield. J Hortic Sci Biotechnol; 2011;86: 319–324. doi: 10.1080/14620316.2011.11512767

[9] Moreira PA, Lins J, Dequigiovanni G, Veasey EA, Clement CR. The Domestication of Annatto (*Bixa orellana*) from *Bixa urucurana* in Amazonia. Econ Bot. 2015;69: 127–135. doi: 10.1007/s12231-015-9304-0

[10] Ducke A. Plantas de cultura pré-colombiana na Amazônia brasileira: notas sobre as espécies ou formas espontâneas que supostamente lhes teriam dado origem. Bol do Inst Agronômico do Norte. 1946;8: 1–24.

[11] Meyer RS, DuVal AE, Jensen HR. Patterns and processes in crop domestication: an historical review and quantitative analysis of 203 global food crops. New Phytol. 2012;196: 29–48. doi: 10.1111/j.1469-8137.2012.04253.x 22889076 10.1111/j.1469-8137.2012.04253.x

[12] Kuntze C. Bixaceae In: Engler A, Prantl K, editors. Die natürlichen Pflanzenfamilien. 2nd ed. Leipzig: Engelmann; 1925 p. 315.

[13] Loveless MD, Hamrick JL. Ecological Determinants of Genetic Structure in Plant Populations. Annu Rev Ecol Syst. Annual Reviews 4139. 1984;15: 65–95. doi: 10.1146/annurev.es.15.110184.000433

[14] Vieira MLC, Santini L, Diniz AL, Munhoz C de F. Microsatellite markers: What they mean and why they are so useful. Genet Mol Biol. Sociedade Brasileira de Genética; 2016;39: 312–328. doi: 10.1590/1678-4685-GMB-2016-0027 27561112 10.1590/1678-4685-GMB-2016-0027 PMC5004837

[15] Valdez-Ojeda R, Hernandez-Stefanoni JL, Aguilar-Espinosa M, Rivera-Madrid R, Ortiz R, Quiros CF. Assessing morphological and genetic variation in Annatto (*Bixa orellana* L.) by sequence-related amplified polymorphism and cluster analysis. HortScience. 2008;43: 2013–2017.

[16] Valdez-Ojeda R, Quiros CF, de Lourdes Aguilar-Espinosa M, Rivera-Madrid R, Aguilar-Espinosa M de L, Rivera-Madrid R. Outcrossing rates in annatto determined by sequence-related amplified polymorphism. Agron J. 2010;102: 1340–1345. doi: 10.2134/agronj2009.0510

[17] Dequigiovanni G, Ramos SLF, Zucchi MI, Bajay MM, Pinheiro JB, Fabri EG, et al Isolation and characterization of microsatellite loci for *Bixa orellana*, an important source of natural dyes. Genet Mol Res. 2014;13 doi: 10.4238/2014.October.31.25 25366801 10.4238/2014.October.31.25

[18] Peterson AT. Predicting the geography of species’ invasions via ecological niche modeling. Q Rev Biol. 2003;78: 419–33. 14737826 10.1086/378926

[19] Coppens d’Eeckenbrugge G, Lacape JM. Distribution and differentiation of wild, feral, and cultivated populations of perennial upland cotton (*Gossypium hirsutum* L.) in Mesoamerica and the Caribbean. PLoS One. 2014;9: e107458 doi: 10.1371/journal.pone.0107458 25198534 10.1371/journal.pone.0107458 PMC4157874

[20] Bonatelli IAS, Perez MF, Peterson AT, Taylor NP, Zappi DC, Machado MC, et al Interglacial microrefugia and diversification of a cactus species complex: phylogeography and palaeodistributional reconstructions for *Pilosocereus aurisetus* and allies. Mol Ecol. 2014;23: 3044–3063. doi: 10.1111/mec.12780 24803224 10.1111/mec.12780

[21] Peres EA, Sobral-Souza T, Perez MF, Bonatelli IAS, Silva DP, Silva MJ, et al Pleistocene Niche Stability and Lineage Diversification in the Subtropical Spider Araneus omnicolor (Araneidae). PLoS One. 2015;10: e0121543 doi: 10.1371/journal.pone.0121543 25856149 10.1371/journal.pone.0121543 PMC4391720

[22] Doyle JJ, Doyle JL. Isolation of Plant DNA from fresh tissue. Focus (Madison). 1990;12: 13–15.

[23] Dequigiovanni G, Ramos SLF, Lopes MTG, Clement CR, Picanço-Rodrigues D, Fabri EG, et al New microsatellite loci for annatto (*Bixa orellana*), a source of natural dyes from Brazilian Amazonia. Crop Breed Appl Biotechnol. 2018;Forthcomin.

[24] Schuelke M. An economic method for the fluorescent labeling of PCR fragments. Nat Biotechnol. Nature Publishing Group; 2000;18: 233–234. doi: 10.1038/72708 10657137 10.1038/72708

[25] Pritchard JK, Stephens M, Donnelly P. Inference of population structure using multilocus genotype data. Genetics. 2000;155: 945–59. 10835412 10.1093/genetics/155.2.945 PMC1461096

[26] Evanno G, Regnaut S, Goudet J. Detecting the number of clusters of individuals using the software STRUCTURE: a simulation study. Mol Ecol. 2005;14: 2611–20. doi: 10.1111/j.1365-294X.2005.02553.x 15969739 10.1111/j.1365-294X.2005.02553.x

[27] El Mousadik A, Petit RJ. High level of genetic differentiation for allelic richness among populations of the argan tree [*Argania spinosa* (L.) Skeels] endemic to Morocco. Theor Appl Genet. Springer-Verlag; 1996;92: 832–839. doi: 10.1007/BF00221895 24166548 10.1007/BF00221895

[28] Wright S. The Interpretation of Population Structure by F-Statistics with Special Regard to Systems of Mating. Evolution. 1965;19: 395 doi: 10.2307/2406450

[29] Vencovsky R. Variance of an estmatve of the outcrossing rate. Rev Bras Genética. 1994;17: 349–351.

[30] Peakall R, Smouse PE. GenAlEx 6.5: genetic analysis in Excel. Population genetic software for teaching and research—an update. Bioinformatics. 2012;28: 2537–2539. doi: 10.1093/bioinformatics/bts460 22820204 10.1093/bioinformatics/bts460 PMC3463245

[31] Keenan K, McGinnity P, Cross TF, Crozier WW, Prodöhl PA. diveRsity: An R package for the estimation and exploration of population genetics parameters and their associated errors. O’Hara RB, editor. Methods Ecol Evol. 2013;4: 782–788. doi: 10.1111/2041-210X.12067

[32] Kamvar ZN, Tabima JF, Grünwald NJ. Poppr: an R package for genetic analysis of populations with clonal, partially clonal, and/or sexual reproduction. PeerJ. PeerJ Inc.; 2014;2: e281 doi: 10.7717/peerj.281 24688859 10.7717/peerj.281 PMC3961149

[33] R Core Team. R: A language and environment for statistical computing [Internet]. Vienna, Austria: R Foundation for Statistical Computing; 2015 Available: https://www.r-project.org/

[34] Saitou N, Nei M. The neighbor-joining method: a new method for reconstructing phylogenetic trees. Mol Biol Evol. 1987;4: 406–25. Available: http://www.ncbi.nlm.nih.gov/pubmed/3447015 doi: 10.1093/oxfordjournals.molbev.a040454 3447015 10.1093/oxfordjournals.molbev.a040454

[35] Felsenstein J. PHYLIP (Phylogeny Inference Package) version 3.6. Seattle: Department of Genome Sciences, University of Washington; 2005.

[36] Cavalli-Sforza LL, Edwards AWF. Phylogenetic Analysis Models and Estimation Procedures. Am J Hum Genet. 1967;19: 233–57. doi: 10.2307/2406616 6026583 PMC1706274

[37] Dieringer D, Schlötterer C. microsatellite analyser (MSA): a platform independent analysis tool for large microsatellite data sets. Mol Ecol Notes. Blackwell Publishing, Ltd; 2003;3: 167–169. doi: 10.1046/j.1471-8286.2003.00351.x

[38] Reif JC, Melchinger AE, Frisch M. Genetical and Mathematical Properties of Similarity and Dissimilarity Coefficients Applied in Plant Breeding and Seed Bank Management. Crop Sci. Crop Science Society of America; 2005;45: 1 doi: 10.2135/cropsci2005.0001

[39] Slatkin M, Barton NH. A Comparison of Three Indirect Methods for Estimating Average Levels of Gene Flow. Evolution. 1989;43: 1349 doi: 10.1111/j.1558-5646.1989.tb02587.x 28564250 10.1111/j.1558-5646.1989.tb02587.x

[40] Jombart T, Ahmed I. adegenet 1.3–1: new tools for the analysis of genome-wide SNP data. Bioinformatics. 2011;27: 3070–3071. doi: 10.1093/bioinformatics/btr521 21926124 10.1093/bioinformatics/btr521 PMC3198581

[41] Phillips SJ, Anderson RP, Schapire RE. Maximum entropy modeling of species geographic distributions. Ecol Modell. 2006;190: 231–259. doi: 10.1016/j.ecolmodel.2005.03.026

[42] Elith J, H. Graham C, P. Anderson R, Dudík M, Ferrier S, Guisan A, et al Novel methods improve prediction of species’ distributions from occurrence data. Ecography (Cop). 2006;29: 129–151. doi: 10.1111/j.2006.0906–7590.04596.x

[43] Hijmans RJ, Cameron SE, Parra JL, Jones PG, Jarvis A. Very high resolution interpolated climate surfaces for global land areas. Int J Climatol. 2005;25: 1965–1978. doi: 10.1002/joc.1276

[44] Fielding AH, Bell JF. A review of methods for the assessment of prediction errors in conservation presence / absence models. Environ Conserv. 1997;24: 38–49. doi: 10.1017/S0376892997000088

[45] Dray S, Dufour A-B. The ade4 Package: Implementing the Duality Diagram for Ecologists. J Stat Softw. 2007;22: 1–20. doi: 10.18637/jss.v022.i04

[46] Dequigiovanni G, Ramos SLF, Alves-Pereira A, Fabri EG, Carvalho PRN, da Silva MG, et al Genetic diversity and structure in a major Brazilian annatto (*Bixa orellana*) germplasm bank revealed by microsatellites and biochemical traits. Genet Resour Crop Evol. 2017; 64; 7: 1775–1788.

[47] Olsen KM, Wendel JF. Crop plants as models for understanding plant adaptation and diversification. Front Plant Sci. Frontiers; 2013;4: 290 doi: 10.3389/fpls.2013.00290 23914199 10.3389/fpls.2013.00290 PMC3729982

[48] Meyer RS, Purugganan MD. Evolution of crop species: genetics of domestication and diversification. Nat Rev Genet. Nature Research; 2013;14: 840–852. doi: 10.1038/nrg3605 24240513 10.1038/nrg3605

[49] Gepts P. Crop Domestication as a Long Term Selection Experiment. Plant Breeding Reviews. 2004 doi: 10.1002/9780470650288.ch1

[50] Ritland K. Extensions of models for the estimation of mating systems using n independent loci. Heredity (Edinb). 2002;88: 221–8. doi: 10.1038/sj.hdy.6800029 11920127 10.1038/sj.hdy.6800029

[51] Rivera-Madrid R, Escobedo-GM RMM, Balam-Galera E, Vera-Ku M, Harries H. Preliminary studies toward genetic improvement of annatto (*Bixa orellana* L.). Sci Hortic. 2006;109: 165–172. doi: 10.1016/j.scienta.2006.03.011

[52] Vilares AS, São José AR, Rebouças TNH, Souza IVB. Estudo da biologia floral de urucuzeiro (*Bixa orellana* L.). Rev Bras Corantes Nat. 1992;1: 101–105.

[53] Dick CW, Hardy OJ, Jones FA, Petit RJ. Spatial Scales of Pollen and Seed-Mediated Gene Flow in Tropical Rain Forest Trees. Trop Plant Biol. 2008;1: 20–33. doi: 10.1007/s12042-007-9006-6

[54] Wright S. Evolution in Mendelian Populations. Genetics. 1931;16: 97–159. Available: http://www.ncbi.nlm.nih.gov/pubmed/17246615 17246615 10.1093/genetics/16.2.97 PMC1201091

[55] Costa A, Guimarães-Dias F, Pérez-Maluf R. Abelhas (Hymenoptera: Apoidea) visitantes das flores de urucum em Vitória da Conquista, BA. Ciência Rural. Universidade Federal de Santa Maria; 2008;38: 534–537. doi: 10.1590/S0103-84782008000200039

[56] Carvalho JFRP de, Robinson IP, Alfenas AC. Isozymic variability in a Brazilian collection of annatto (*Bixa orellana* L.). Pesqui Agropecuária Bras. Embrapa Informação Tecnológica; 2005;40: 653–660. doi: 10.1590/S0100-204X2005000700005

[57] Medina AM, Michelangeli CC, Ramis CM, Díaz AJ. Caracterización morfológica de frutos de onoto (*Bixa orellana* L.) y su correspondencia con patrones de proteínas e isoenzimas. Acta Científica Venez. 2001;52: 14–23. 11510423

[58] de Menezes IPP, Gaiotto FA, Hoffmann LV, Ciampi AY, Barroso PAV. Genetic diversity and structure of natural populations of *Gossypium mustelinum*, a wild relative of cotton, in the basin of the De Contas River in Bahia, Brazil. Genetica. 2014;142: 99–108. doi: 10.1007/s10709-014-9757-6 24473734 10.1007/s10709-014-9757-6

[59] INPE. Monitoring of the Brazilian Amazonian forest by satellite, 2000–2001 [Internet]. São José dos Campos, SP, Brazil: Instituto Nacional de Pesquisas Espaciais; 2002 Available: http://www.inpe/br/informacoes_Eventos/amazonia.htm

[60] Wiens JJ. The niche, biogeography and species interactions. Philos Trans R Soc Lond B Biol Sci. 2011;366: 2336–2350. doi: 10.1098/rstb.2011.0059 21768150 10.1098/rstb.2011.0059 PMC3130432

[61] Wang T, Wang Z, Xia F, Su Y. Local adaptation to temperature and precipitation in naturally fragmented populations of *Cephalotaxus oliveri*, an endangered conifer endemic to China. Sci Rep. Nature Publishing Group; 2016;6: 25031 doi: 10.1038/srep25031 27113970 10.1038/srep25031 PMC4844950

[62] Manel S, Poncet BN, Legendre P, Gugerli F, Holderegger R. Common factors drive adaptive genetic variation at different spatial scales in *Arabis alpina*. Mol Ecol. 2010;19: 3824–3835. doi: 10.1111/j.1365-294X.2010.04716.x 20723057 10.1111/j.1365-294X.2010.04716.x

[63] Reusch TBH, Wood TE. Molecular ecology of global change. Mol Ecol. 2007;16: 3973–92. doi: 10.1111/j.1365-294X.2007.03454.x 17894755 10.1111/j.1365-294X.2007.03454.x

[64] Clement CR, Rodrigues DP, Alves-Pereira A, Mühlen GS, De Cristo-Araújo M, Moreira PA, et al Crop domestication in the upper Madeira River basin. Bol do Mus Para Emilio GoeldiCiencias Humanas. 2016;11: 193–205. doi: 10.1590/1981.81222016000100010

[65] Levis C, Costa FRC, Bongers F, Peña-Claros M, Clement CR, Junqueira AB, et al Persistent effects of pre-Columbian plant domestication on Amazonian forest composition. Science. 2017;355: 925–931. doi: 10.1126/science.aal0157 28254935 10.1126/science.aal0157

[66] Piperno DR, Pearsall DM. The origins of agriculture in the lowland neotropics Academic Press; 1998 Available: http://www.sciencedirect.com/science/book/9780125571807

[67] Walker JH. The Llanos de Mojos The Handbook of South American Archaeology. New York, NY: Springer New York; 2008 pp. 927–939. doi: 10.1007/978-0-387-74907-5_46

[68] Clement CR. 1492 and the loss of amazonian crop genetic resources. I. The relation between domestication and human population decline. Econ Bot; 1999;53: 188–202. doi: 10.1007/BF02866498

[69] Erickson C. Archaeological methods for the study of ancient landscapes of the Llanos de Mojos in the Bolivian Amazon In: Stahl P, editor. Archaeology in the lowland American tropics: Current analytical methods and applications. Cambridge: Cambridge University Press; 1995 pp. 66–95.

[70] Leal F, Clavijo CM. Acerca de la história, taxonomia, botánica y usos de *Bixa orellana* L. Rev Unell Cienc Tec. 2010;1: 78–86.

[71] Barthem R, Goulding M, Forsberg B, Canas C, Ortega H. Aquatic Ecology of the Rio Madre de Dios. Scientific bases for Andes Amazon Headwaters. (ACCA). Lima, Peru: Gráfica Biblos; 2003.

[72] Osher L., Buol S. Relationship of soil properties to parent material and landscape position in eastern Madre de Dios, Peru. Geoderma. 1998;83: 143–166. doi: 10.1016/S0016-7061(97)00133-X

[73] Stefano R, Aymard G, Riina R, Huber O. Flora and Vegetation of the Venezuelan Llanos: a review. In: Pennington R, Lewis G, Ratter J, editors. Neotropical Savannas and Seasonally Dry Forests Plant Diversity, Biogeography, and Conservation. 1st ed. New York; 2006. pp. 96–118.

