# Supplementary material for: Highly structured genetic diversity of Bixa orellana var. urucurana, the wild ancestor of annatto, in Brazilian Amazonia
Source: PLoS One. 2018 Jun 6;13(6):e0198593. doi: 10.1371/journal.pone.0198593 (PMC5991381; doi:10.1371/journal.pone.0198593)
Supplement: S2 Table — (DOCX) [file pone.0198593.s002.docx]

**S2 Table. Factor loadings of Principal component analysis (rotation) on a set of 19 bioclimatic variables retained for their contribution to the model of distribution (higher values in bold characters).**

| Variable | PC1 (71%) | PC2 (20%) | PC3 (6%) | PC4 (2%) |
| --- | --- | --- | --- | --- |
| BIO1 = Annual Mean Temperature | -0.0003 | 0.0023 | 0.0002 | -0.0003 |
| BIO2 = Mean Diurnal Range | -0.0005 | -0.0011 | -0.0026 | -0.0017 |
| BIO3 = Isothermality (BIO2/BIO7) | 0.0032 | 0.0008 | 0.0117 | 0.0080 |
| BIO4 = Temperature Seasonality | -0.0046 | 0.0028 | -0.0271 | -0.0600 |
| BIO5 = Max Temperature of Warmest Month | -0.0007 | 0.0025 | -0.0025 | -0.0013 |
| BIO6 = Min Temperature of Coldest Month | 0.0006 | 0.0042 | 0.0026 | 0.0022 |
| BIO7 = Temperature Annual Range | -0.0013 | -0.0017 | -0.0052 | -0.0034 |
| BIO8 = Mean Temperature of Wettest Quarter | -0.0004 | 0.0012 | 0.0000 | -0.0010 |
| BIO9 = Mean Temperature of Driest Quarter | 0.0000 | 0.0037 | 0.0006 | 0.0022 |
| BIO10 = Mean Temp. of Warmest Quarter | -0.0003 | 0.0026 | -0.0003 | -0.0006 |
| BIO11 = Mean Temperature of Coldest Quarter | -0.0002 | 0.0024 | 0.0004 | 0.0007 |
| BIO12 = Annual Precipitation | **0.8757** | -0.1919 | -0.1250 | **0.3052** |
| BIO13 = Precipitation of Wettest Month | 0.0759 | 0.0567 | -0.2527 | -0.1002 |
| BIO14 = Precipitation of Driest Month | 0.0581 | -0.0527 | 0.1833 | 0.0580 |
| BIO15 = Precipitation Seasonality | **-0.0190** | 0.0251 | -0.0670 | -0.0363 |
| BIO16 = Precipitation of Wettest Quarter | 0.2129 | 0.1468 | **-0.6377** | -0.1877 |
| BIO17 = Precipitation of Driest Quarter | 0.1946 | -0.1584 | **0.5789** | 0.2289 |
| BIO18 = Precipitation of Warmest Quarter | 0.2106 | **-0.4472** | 0.1891 | **-0.8459** |
| BIO19 = Precipitation of Coldest Quarter | 0.3099 | **0.8425** | 0.3224 | -0.2921 |
