# Supplementary figures and images for: Highly structured genetic diversity of Bixa orellana var. urucurana, the wild ancestor of annatto, in Brazilian Amazonia
Source: PLoS One. 2018 Jun 6;13(6):e0198593. doi: 10.1371/journal.pone.0198593 (PMC5991381; doi:10.1371/journal.pone.0198593)

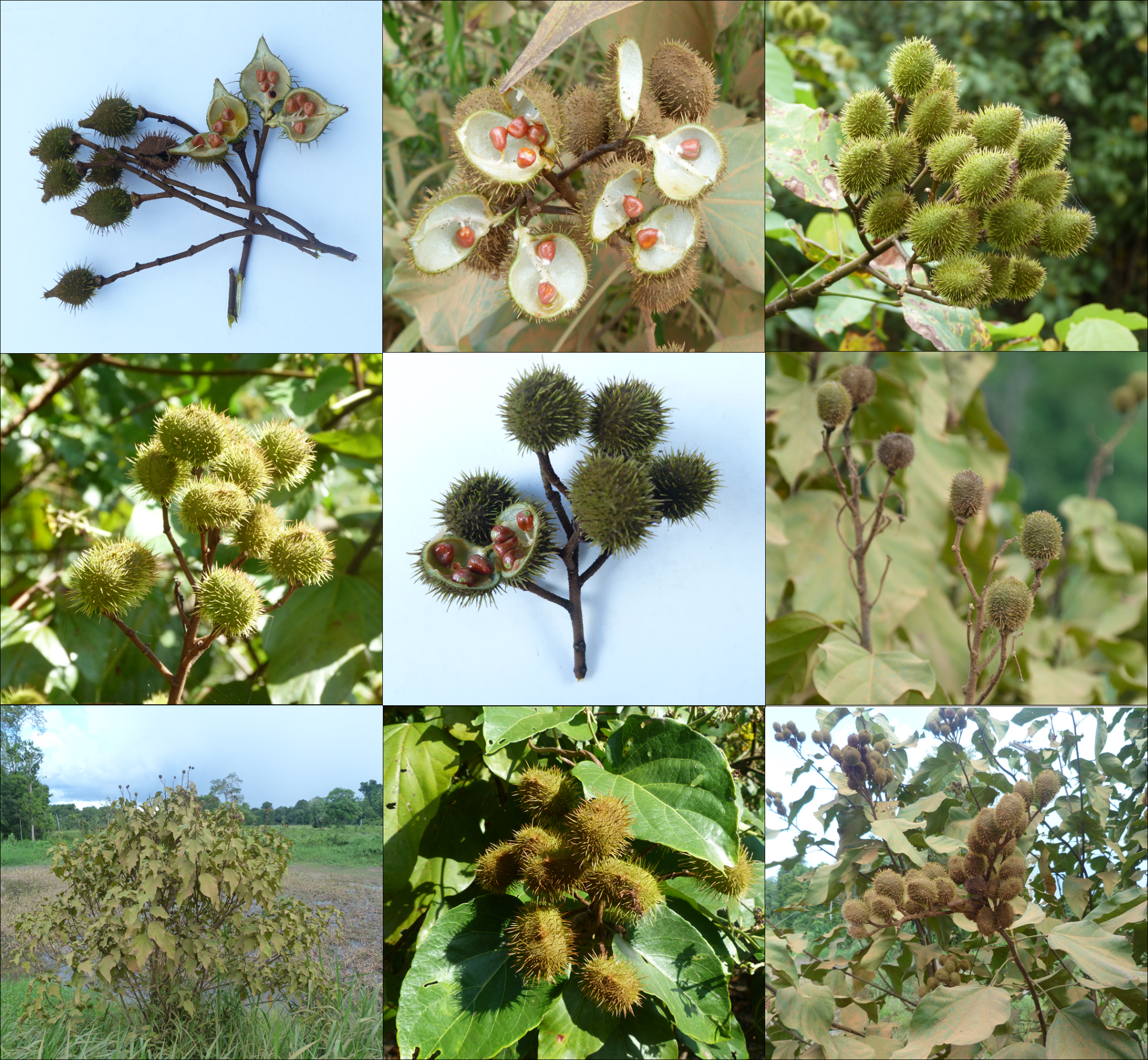

Supplement: S1 Fig — (TIFF) [file pone.0198593.s003.tiff]

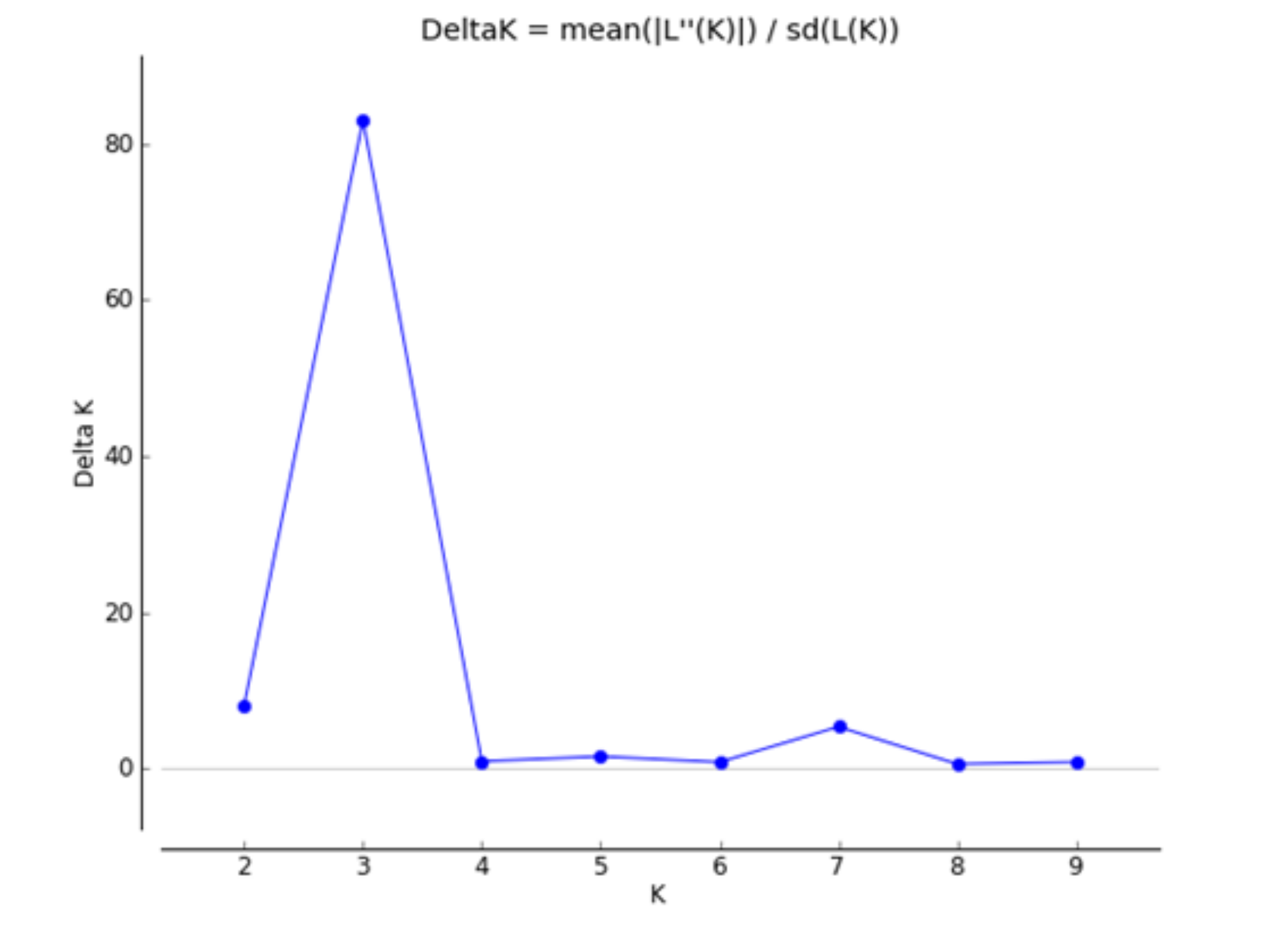

Supplement: S2 Fig — (TIFF) [file pone.0198593.s004.tiff]

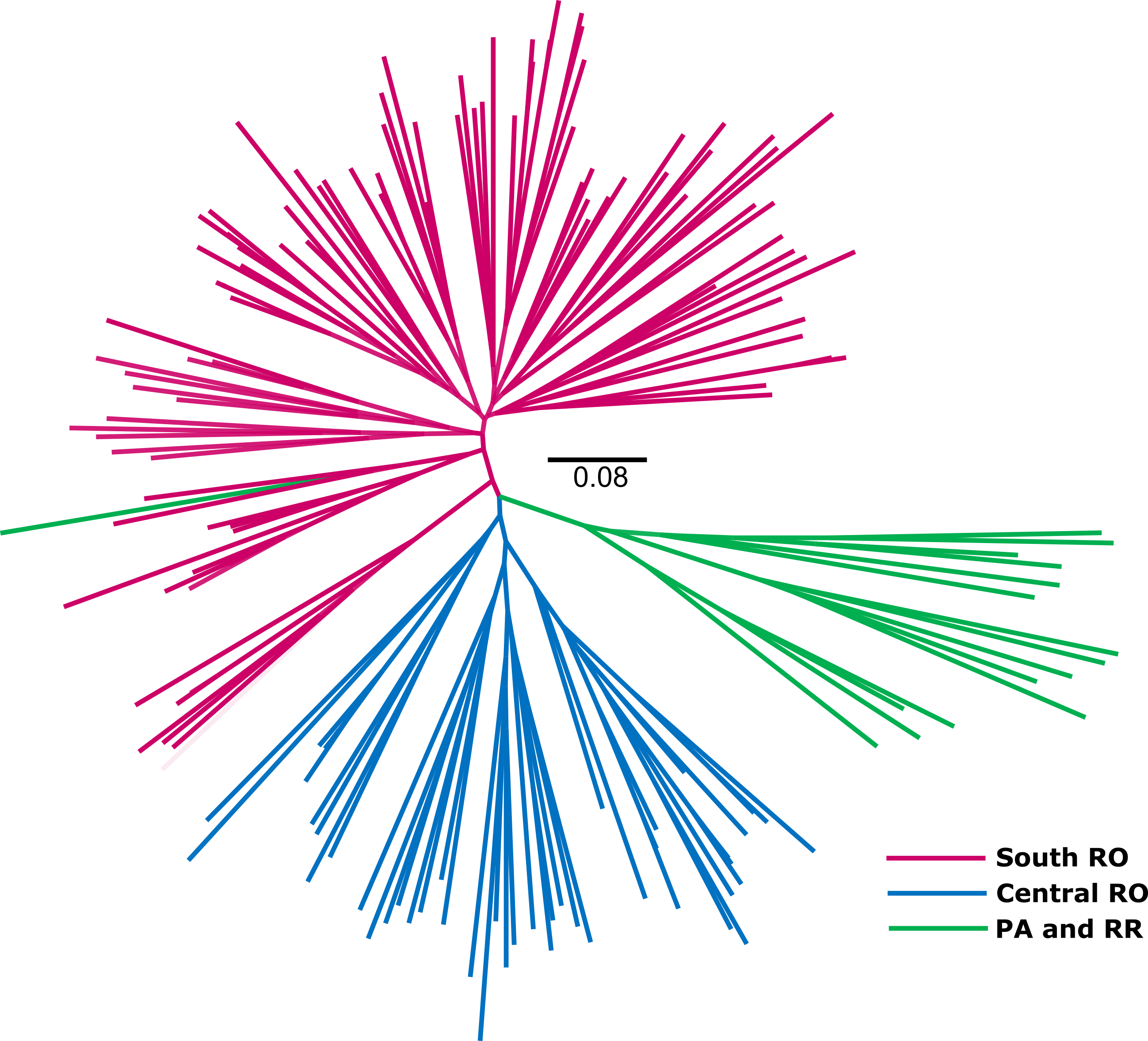

Supplement: S3 Fig — Branches are colored according to the Structure simulation for K = 3. (TIFF) [file pone.0198593.s005.tiff]
